# Protection of insect neurons by erythropoietin/CRLF3-mediated regulation of pro-apoptotic acetylcholinesterase

**DOI:** 10.1038/s41598-022-22035-0

**Published:** 2022-11-03

**Authors:** Debbra Y. Knorr, Kristin Schneider, Luca Büschgens, Jan Förster, Nadine S. Georges, Bart R. H. Geurten, Ralf Heinrich

**Affiliations:** Department of Cellular Neurobiology, Johann-Friedrich-Blumenbach-Institute for Zoology and Anthropology, Georg-August-University Göttingen, Göttingen, Germany

**Keywords:** Apoptosis, Cell signalling, Extracellular signalling molecules

## Abstract

Cytokine receptor-like factor 3 (CRLF3) is a conserved but largely uncharacterized orphan cytokine receptor of eumetazoan animals. CRLF3-mediated neuroprotection in insects can be stimulated with human erythropoietin. To identify mechanisms of CRLF3-mediated neuroprotection we studied the expression and proapoptotic function of acetylcholinesterase in insect neurons. We exposed primary brain neurons from *Tribolium castaneum* to apoptogenic stimuli and dsRNA to interfere with acetylcholinesterase gene expression and compared survival and acetylcholinesterase expression in the presence or absence of the CRLF3 ligand erythropoietin. Hypoxia increased apoptotic cell death and expression of both acetylcholinesterase-coding genes *ace-1* and *ace-2*. Both *ace* genes give rise to single transcripts in normal and apoptogenic conditions. Pharmacological inhibition of acetylcholinesterases and RNAi-mediated knockdown of either *ace-1* or *ace-2* expression prevented hypoxia-induced apoptosis. Activation of CRLF3 with protective concentrations of erythropoietin prevented the increased expression of acetylcholinesterase with larger impact on *ace-1* than on *ace-2*. In contrast, high concentrations of erythropoietin that cause neuronal death induced *ace-1* expression and hence promoted apoptosis. Our study confirms the general proapoptotic function of AChE, assigns a role of both *ace-1* and *ace-2* in the regulation of apoptotic death and identifies the erythropoietin/CRLF3-mediated prevention of enhanced acetylcholinesterase expression under apoptogenic conditions as neuroprotective mechanism.

## Introduction

Erythropoietin (Epo) is a helical cytokine generally known for its functions in vertebrate erythropoiesis, where it protects erythrocyte progenitor cells from apoptosis^1–3^. Local production and cytoprotective functions of Epo have been discovered in various vertebrate tissues^4–9^. Cell-protective concentrations of Epo vary between cell types, species and types of insult^10–13^. Concerning neurons from various species, optimum-type concentration-responses have been reported in which high Epo concentrations not only lack the protective effects but rather exert cytotoxic effects leading to increased cell death compared with untreated control cells^14–19^. Reduced protective effects with Epo concentrations above the optimum have been explained by reduced cell surface localization of EpoR^20,21^, inhibition of EpoR-initiated transduction^22^ and prevention of EpoR homo-dimerization due to saturation of high-affinity binding sites of EpoR monomers^23^. An explanation for toxic effects of very high concentrations of Epo is currently lacking for EpoR, CRLF3 or other potential alternative Epo receptors. Epo-mediated cell protection typically relies on upregulation of anti-apoptotic proteins following phosphorylation of JAK associated with Epo receptors (reviewed in Ref.^24^). Nonetheless, a clear picture of Epo-mediated anti-apoptotic effects remains elusive.

Most insects express the phylogenetically conserved orphan cytokine receptor CRLF3 (cytokine receptor-like factor 3). CRLF3 belongs to group 1 of the prototypic class I cytokine receptors which also includes the classical erythropoietin receptor EpoR^25,26^. Erythropoietin signalling initiates neuroprotective processes in the mammalian nervous system^5,7,27,28^ by activating homodimeric EpoR and/or alternative Epo receptors^29–31^. *EPO* and *EPOR* are widely but exclusively expressed in vertebrate species and therefore absent in insects. Nonetheless, recombinant human Epo (rhEpo) protects locust and beetle neurons from toxin- and hypoxia-induced apoptosis by activating partially identical intracellular transduction pathways as in mammalian cells^10,19,32^. CRLF3 was identified as the insect neuroprotective receptor for rhEpo and EV-3, a splice variant of human Epo with neuroprotective properties that cannot activate homodimeric EpoR^19,33,34^. Signalling via an unknown Epo-like cytokine and CRLF3 seems to represent an ancient cell-protective system that secures neuron and other cells’ survival and maintenance of tissue functionality under unfavourable physiological conditions.

In addition to its function at cholinergic synapses (Zhang et al*.*^35^), acetylcholinesterase (AChE) contributes to multiple processes including cellular adhesion, cell growth, cell differentiation, amyloid fiber assembly and apoptosis^36–39^. Altered presence and functions of AChE are associated with various degenerative diseases including Alzheimer’s disease, Parkinson’s disease and cancer in various tissues^40–43^. Mammalian species express three major AChE splice variants from a single gene locus, that differ in their carboxy-terminal domains which determine localisation and interactions with other proteins^37,44–46^. Splice variants include the synaptic AChE (AChE-S), erythrocytic AChE (AChE-E) and the soluble read-through variant (AChE-R). Apoptogenic physiological stress enhances intracellular AChE levels in various mammalian tissues (including brain, retina, kidney, endothelial cells, bone, myoblasts) and cell lines (including PC12, neuroblastoma, HeLa cells) (reviewed by Refs.^37,47^. Increased levels of AChE sensitize cells to induce apoptosis upon exposure to pathogenic or physiologically challenging conditions^48^. Absence or catalytic inactivation of AChE have been correlated with reduced sensitivity to apoptogenic stimuli and reduced cell death in various cell types including neurons^42,49^. Identified pro-apoptotic mechanisms of mammalian AChE include the facilitation of apoptosom formation following mitochondrial cytochrome c release^35,37,50,51^ and the degradation of DNA following nuclear translocation during apoptosis^52^.

In contrast to mammals, most insects possess two distinct genes (*ace-1* and *ace-2*) coding for different AChE proteins^53–55^. Depending on the species, either AChE-1 or AChE-2 mediates the canonical, synaptic functions of the enzyme^53,55–57^, while functions of the other protein remain largely uncharacterised^53,55,56,58,59^. Our previous studies identified a pro-apoptotic function of AChE in neurons of the migratory locust *Locusta migratoria*^60^ that parallels the role of AChE mammalian apoptosis. We demonstrated that *Lm-ace-1* transcript levels increased under hypoxic conditions in vivo and that pharmacological inhibition of AChE prevented hypoxia-induced apoptotic death of locust primary neurons. This indicates a link between AChE-1 presence/activity and apoptotic cell death in insects^60^. Since genetic information in locusts is scarce, sequence information is only available for *Lm-ace-1* but not for *Lm-ace-2.* This prevents the investigation of differential functions of *ace-1* and *ace-2* in apoptosis and other processes in locust species. The red flour beetle *Tribolium castaneum* expresses different *ace* transcripts and AChE proteins from two genes, with cholinergic functions accounted to *Tc-ace-1* and other, incompletely identified functions in developmental processes of *Tc-ace-2*^52,54,57^. Given that sequences for both *Tc-ace-1* and *Tc-ace-2* are available and protocols for in vitro studies with primary neurons were previously established, we decided to analyse the differential involvement of the two *ace* genes and AChE proteins in *T. castaneum* apoptosis.

The present study explores the possibility that previously reported neuroprotective functions of Epo/CRLF3 are mediated through prevention of pro-apoptotic AChE expression. With experiments on whole pupae and primary neuron cultures from *T. castaneum*, we explore the differential expression of *Tc-ace-1* and *Tc-ace-2* under apoptogenic conditions (hypoxia) and their contribution to the progress of apoptosis. We link the previously reported neuroprotective effect of rhEpo-mediated CRLF3 activation in insects with the reduced expression of pro-apoptotic AChE. While neuroprotective concentrations of rhEpo prevented overexpression of *ace-1* under apoptogenic conditions, toxic concentrations of rhEpo increased *ace-1* expression. Given the known pro-apoptotic functions of AChE in mammalian neurons (and other cells), Epo-mediated neuroprotection via EpoR and/or alternative Epo receptors may also rely on negative regulation of *ACHE* expression.

## Results

### Involvement of ace in *T. castaneum* apoptosis

**Figure 1:**
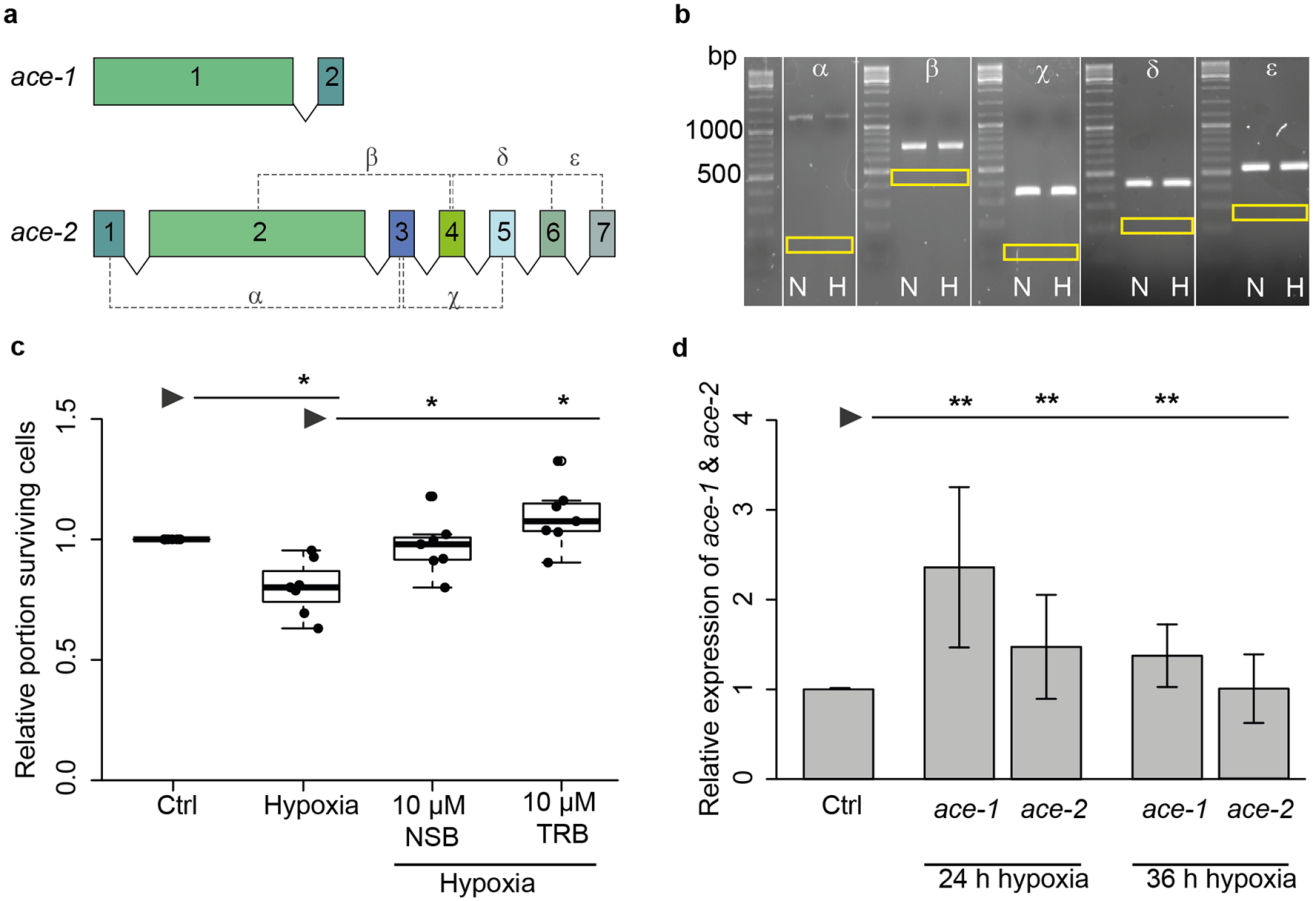
AChE/*ace* in *Tribolium castaneum* neurons. (**a**) Schematic representation of *T. castaneum Tc-ace-1* and *Tc-ace-2* genes. Exons are represented as boxes with sizes corresponding to exon length. Introns are represented as lines that do not depict intron length. For splice variant identification exon spanning primers were designed as depicted by dashed lines. (**b**) RT-PCR analysis for splice variant identification in brains of untreated pupae (*N* normoxia) and pupae after 24 h hypoxia-exposure (< 0.3% O_2_) (*H* hypoxia). Yellow boxes show the expected band size if the sandwiched exon was spliced out. Greek letters above panels correspond to primer pairs depicted in (**a**). (**c**) Relative cell survival of *T. castaneum* primary neurons in vitro. Cells were cultured for 5 days before being exposed to hypoxia (< 0.3% O_2_; 36 h). Two groups were treated with AChE inhibitors NSB or TRB (each 10 µM) for the entire culturing period. Cell survival was analysed by DAPI staining and normalized to the untreated control of each experiment (set to 1). Hypoxia significantly reduces cell survival. Inhibition of AChE with either NSB or TRB completely prevent hypoxia-induced apoptosis. n = 7, 73.118 cells analyzed. Statistics with pairwise permutation test and Benjamini–Hochberg correction. (**d**) qPCR analysis of *Tc-ace-1* and *Tc-ace-2* transcript expression in *T. castaneum* brains after hypoxia-exposure. 24 h hypoxia significantly increases transcript levels of both *Tc-ace-1* and *Tc-ace-2*. After 36 h hypoxia only *Tc-ace-1* transcript levels remain significantly elevated compared to normoxic control animals. Expression of each *ace* was compared and normalized to its expression in the respective control group. Data represent the mean ± SD. n = 3, *rps3* and *rps18* were used as housekeeping genes. Statistics with pairwise permutation test and Benjamini–Hochberg correction for multiple comparison. *p < 0.05, **p < 0.01.

Before studying the role of AChE in the neuronal apoptosis of *T. castaneum* we explored the possibility of multiple splice variants from the two *ace* genes. While *ace-1* includes only two exons making alternative transcripts rather unlikely, *ace-2* consists of seven exons carrying the potential for multiple different splice variants (Fig. 1a). We designed primers spanning central regions of various pairs of *ace-2* exons (indicated in Fig. 1a) in order to detect potential splice variants in the present transcripts. Transcripts were analysed in brains of untreated pupae and brains of pupae after 24 h exposure to hypoxia (< 0.3% O_2_). RT-PCR analysis revealed no alternative splicing products of *ace-2*, neither in normoxic control nor in hypoxia-treated pupae (Fig. 1b). All detected PCR products included the exon that was interspersed between the two exons targeted by the primers. Hence, all PCR products were clearly larger than expected if the sandwiched exon was spliced out (size of potential PCR product from alternate transcript indicated by yellow boxes in Fig. 1b). The results are in line with the existence of only one transcript that includes all seven exons in normal and hypoxia-challenged *T. castaneum* brains. This also agrees with results of a previous study that detected multiple forms of AChE-1 and/or AChE-2 in some insect species but not in *T. castaneum*^52^.

We previously demonstrated that pharmacological inhibition of AChE rescues primary cultured locust neurons from hypoxia-induced apoptosis and that the induction of specific apoptotic markers (e.g. TUNEL staining) correlates with changes in chromatin structure visualized by DAPI labelling^60^. Following a similar protocol, primary neuron cultures from *T. castaneum* were exposed to hypoxic conditions (< 0.3% O_2_) for 36 h. Hypoxia-exposure reduced the median relative survival of cultured neurons (0.8) in comparison to normoxic control cultures (normalized to 1.0; Fig. 1c; p = 0.013). Hypoxia-induced cell death was completely prevented in the presence of 10 µM of the two AChE inhibitors neostigmine bromide (NSB; median relative survival 0.98; p = 0.044) and territrem B (TRB; median relative survival 1.1; p = 0.013). Neuron survival in hypoxia was significantly increased by both AChE inhibitors compared to untreated hypoxic cultures reaching the same level as the normoxic control cultures.

Expression of *Tc-ace-1* and *Tc-ace-2* under apoptogenic conditions was studied by qPCR in brains of *T. castaneum* pupae following hypoxia-exposure (< 0.3% oxygen) for 24 and 36 h. Expression levels of either *ace* were normalized to their expression in respective untreated controls. 24 h hypoxia significantly increased transcript levels of both *Tc-ace-1* (2.36-fold ± 0.8 SD; p = 0.0018) and *ace-2* (1.47-fold ± 0.6 SD; p = 0.0089) compared to brains of control animals in normoxic atmosphere (Fig. 1d). Prolonging the hypoxic period to 36 h reduced high expression levels detected after 24 h. While *Tc-ace-1* expression remained significantly elevated (1.37-fold ± 0.3 SD; p = 0.0089), *Tc-ace-2* transcript levels were no longer different from controls kept under normoxic conditions (1.02-fold ± 0.4 SD; p = 0.64). The results presented in Fig. 1c,d indicate a pro-apoptotic involvement of both *T. castaneum ace* genes in hypoxia-induced neuronal apoptosis*.*

**Figure 2:**
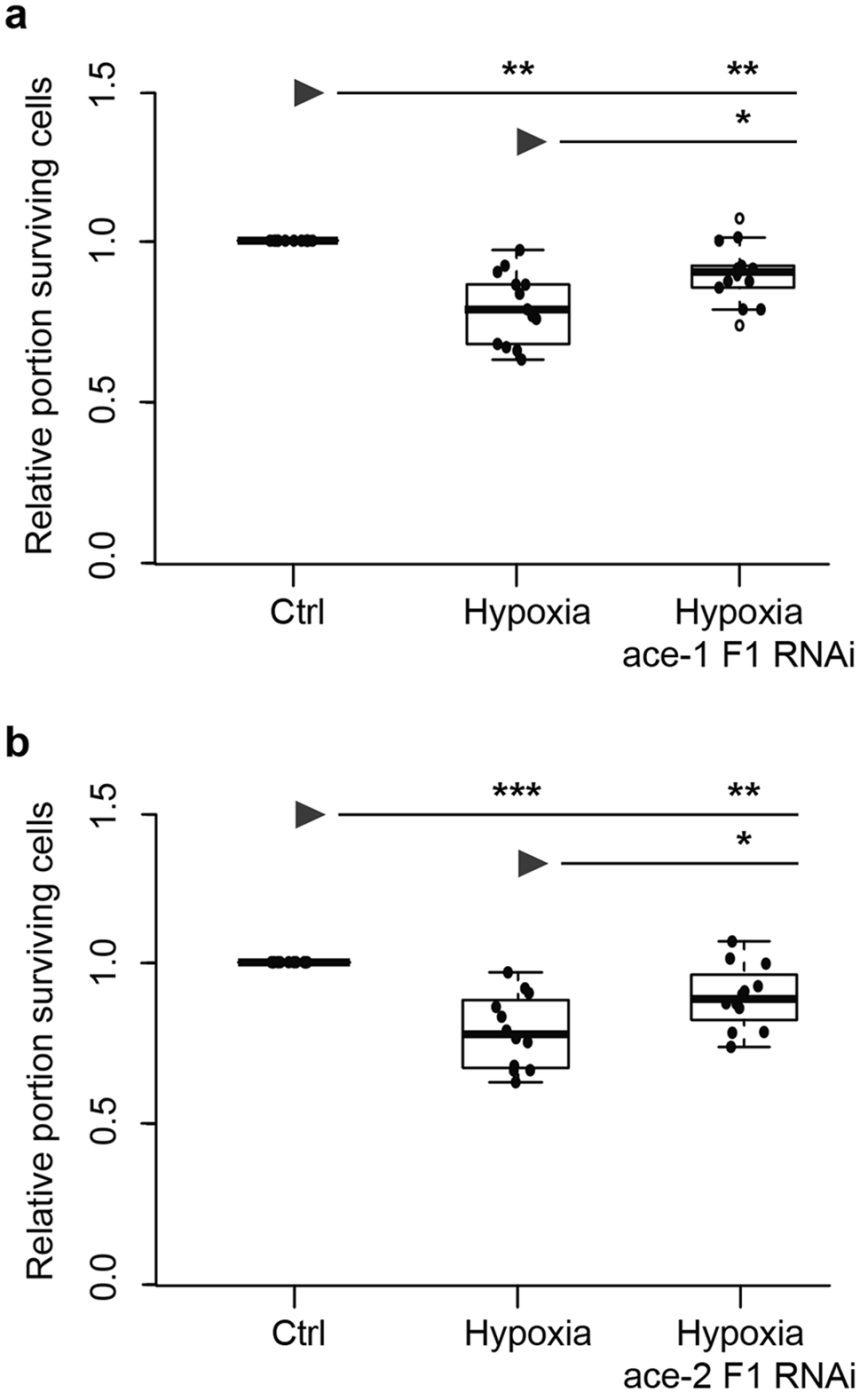
Relative survival of hypoxia-exposed *T. castaneum* primary neurons after RNAi-mediated knock down of *ace-1* and *ace-2* expression. Primary cell cultures were maintained for 5 days in vitro with addition of dsRNA before being exposed to hypoxia (< 0.3% O_2_) for 36 h. Cell survival was analysed by DAPI staining and normalized to the respective untreated control of each experiment (set to 1). (**a,b**) Hypoxia significantly decreased neuron survival in comparison to untreated control cultures. (**a**) Knockdown of *ace-1* using fragment 1 significantly increased relative survival of hypoxia-exposed primary neurons. Cell survival is yet significantly lower in comparison to normoxic controls. Empty circles depict outliers. n = 8, 156.006 cells analyzed. (**b**) Knockdown of *ace-2* with fragment 1 partially rescues neurons from hypoxia-induced apoptosis. However, relative cell survival is still significantly lower in comparison to normoxic control cultures. n = 12, 145.894 cells analyzed. Statistics with pairwise permutation test and Benjamini–Hochberg correction for multiple comparisons. Significant differences are indicated by asterisks (*p < 0.05; **p < 0.01; ***p < 0.001).

In order to assess the individual contributions of *Tc-ace-1* and *Tc-ace-2* to hypoxia-induced apoptosis in *T. castaneum* we inhibited the production of the respective AChE proteins by RNA interference in primary cultured brain neurons before subjecting them to hypoxia (< 0.3% O_2_; 36 h). Effectiveness of knock downs was validated by qPCR analysis (see Supporting Information Data). Neuron survival was compared between normoxic control cultures, hypoxia-exposed cultures and hypoxia-exposed cultures after dsRNA-mediated knockdown of either *Tc-ace-1* or *Tc-ace-2* expression. For each *ace* gene two dsRNA fragments that target non-overlapping regions of the respective transcript, were designed and knockdown was induced by soaking RNAi as described previously^19,60^. Figure 2 depicts data from experiments with the respective fragment 1 to knock down *Tc-ace-1* and *Tc-ace-2* expression (Data from experiments with fragment 2 are provided with the supporting information (Suppl. Fig. S1).

Hypoxia significantly reduced relative neuron survival compared with normoxic control cultures in both experimental series (Fig. 2a: median relative survival 0.80, p = 0.0013; Fig. 2b: median relative survival 0.78, p = 0.00015). Knock down of *Tc-ace-1* with fragment 1 significantly increased relative neuron survival in hypoxia-exposed cultures (median relative survival 0.97; p = 0.013), however this rescue was incomplete without reaching survival levels as in normoxic control cultures (Fig. 2a; p = 0.0035). Knock down of *Tc-ace-1* expression with fragment 2 elevated median relative neuron survival in hypoxia-exposed cultures from 0.77 to 0.82 (Suppl. Fig. S1 in Supporting Information). RNAi-mediated suppression of *Tc-ace-2* expression with fragment 1 significantly increased neuron survival in hypoxia-exposed cultures (median relative survival 0.89; p = 0.022) (Fig. 2b). Similar results were obtained after knock down of *Tc-ace-2* expression with fragment 2 which increased median neuron survival in hypoxia from 0.84 to 0.94 (Suppl. Fig. S1b in supporting information, p = 0.044). However, interference with *Tc-ace-2* expression with either fragment was not sufficient to increase cell survival in hypoxia to the levels of normoxic cultures (F1 p = 0.0022; F2 p = 0.044). In summary, dsRNA-mediated interference with *Tc-ace-1* and *Tc-ace-2* expression for 5 days prior to hypoxia-exposure partially rescues *T. castaneum* primary neurons from hypoxia-induced apoptosis.

### rhEpo prevents hypoxia-induced apoptosis and elevated ace expression

Previous studies reported anti-apoptotic effects of rhEpo on locust and beetle neurons^19,32^ whereas AChE was associated with pro-apoptotic activity (Ref.^60^; this study). In order to evaluate a potential convergence of these pro- and anti-apoptotic pathways we combined rhEpo and AChE-inhibitor treatment of hypoxia-exposed neurons and studied potential regulatory effects of rhEpo on *ace* expression in *T. castaneum* primary neuron cultures.

**Figure 3:**
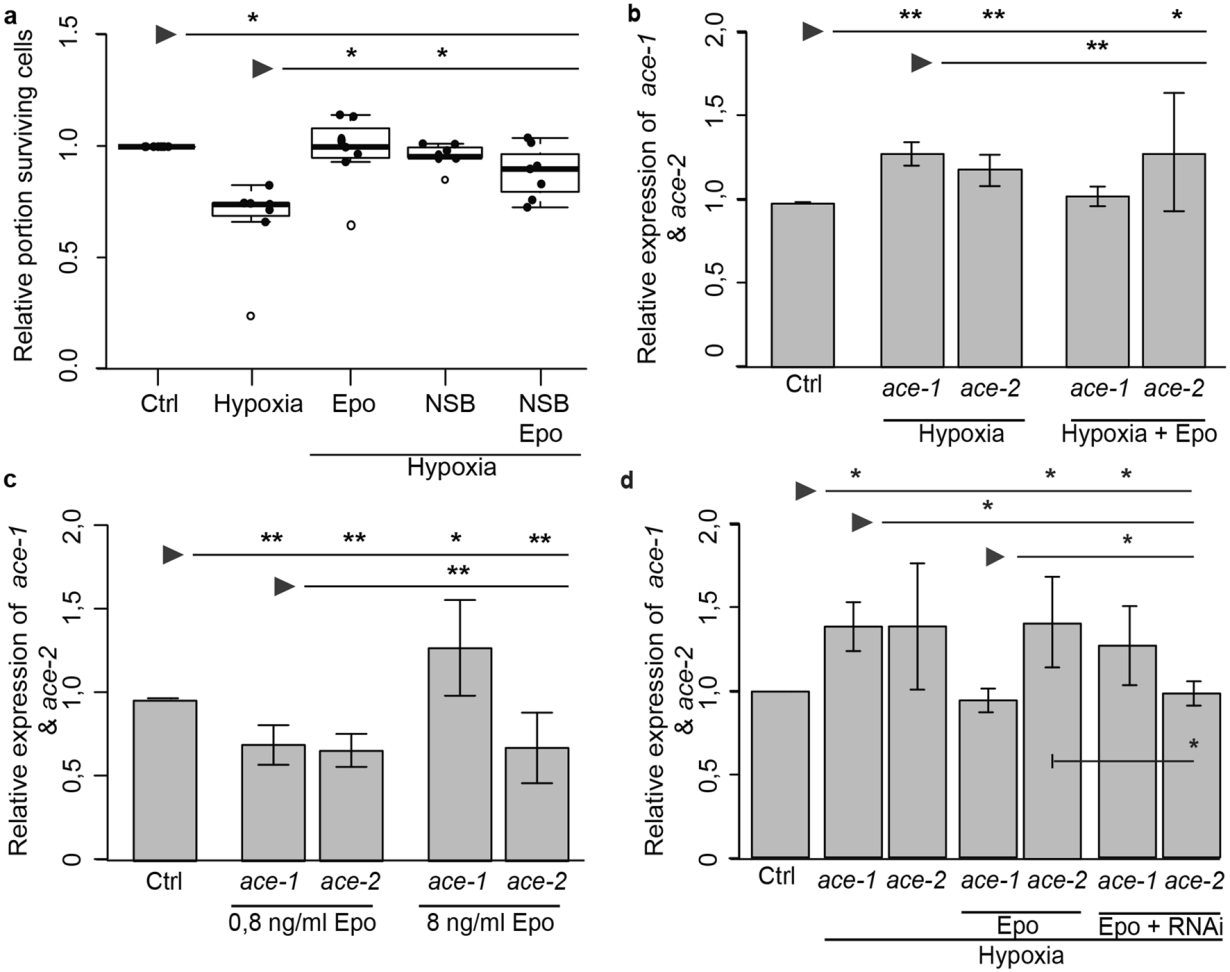
rhEpo-mediated regulation of neuron survival and *ace* expression in hypoxia-exposed *T. castaneum* primary neuron cultures. Cultures were exposed to hypoxic conditions (< 0.3% O_2_ for 36 h) and treated with 10 µM NSB (entire in vitro period) or/and 0.8 ng/ml rhEpo (starting 12 h before start of hypoxic period). (**a**) Relative survival of primary neurons was normalized to respective untreated normoxic controls (Set to 1). Cells were maintained for 5 days, with two groups receiving NSB (10 µM) over the entire culturing period. Epo (0.8 ng/ml) was added 12 h before hypoxia exposure. Cell survival was analysed via DAPI staining. Hypoxia significantly decreased neuron survival. rhEpo and NSB prevent hypoxia-induced cell death and increase survival to the level of normoxic controls. Combined treatment with rhEpo/NSB increases neuron survival in hypoxia-exposed cultures without reaching significance level. Empty circles depict outliers. n = 7, 112.114 cells analyzed. (**b**) qPCR analysis of *Tc-ace-1* and *Tc-ace-2* expression in *T. castaneum* primary neuron cultures. Hypoxia increases expression of both *Tc-ace-1* (1.2 ± 0.2 SD) and *Tc-ace-2* (1.33 ± 0.3 SD). rhEpo inhibited the hypoxia-induced overexpression of *Tc-ace-1* (1.05 ± 0.2 SD) but not of *Tc-ace-2* (1.2 ± 0.1 SD). n = 3. (**c**) Primary neuron cultures were depleted of serum on day 3 in vitro and exposed to protective and toxic concentrations of rhEpo for 48 h starting on day 5 in vitro. *T. castaneum:* 0.8 ng/ml rhEpo (= protective concentration) decrease *Tc-ace-1* and *Tc-ace-2* transcript levels to 0.71-fold (± 0.1 SD) and 0.69-fold (± 0.1 SD) respectively. 8 ng/m-rhEpo (= toxic concentration) increased *Tc-ace-1* transcript levels (1.33-fold ± 0.3 SD) but reduced *Tc-ace-2* transcript levels (0.71-fold ± 0.2 SD) in comparison to untreated controls. n = 3. **(d**) Epo mediated regulation of *ace* expression involves CRLF3. *T. castaneum* primary brain cell cultures were treated with dsRNA targeting *CRLF3* for 5 days. Cells were treated with 0.8 ng/ml rhEpo on day 5 and exposed to hypoxia 12 h later for 36 h. Hypoxia significantly increased *ace-1* gene expression (1.39 ± 0.15 SD), which was prevented when neurons received Epo before hypoxia (0.95 ± 0.07 SD). RNAi-mediated knock down of *CRLF3* expression prevented Epo-mediated reduction of *ace-1* transcript levels in hypoxia-exposed cultures without significant difference to hypoxia-only exposed cultures (1.28 ± 0.24 SD compared with 1.39 ± 0.15 SD). *Ace-2* expression levels were elevated in hypoxia, which was not prevented by rhEpo. Knockdown of CRLF3 expression reduced ace-2 transcript levels of hypoxia/Epo-exposed neurons to the level of normoxic controls. n = 3. For (**b–d**) Expression of each *ace* transcript was compared and normalized to expression within the respective control group. Statistics with pairwise permutation test and Benjamini–Hochberg correction. Significant differences are indicated by asterisks (*p < 0.05; **p < 0.01).

In primary neuron cultures of *T. castaneum* hypoxia-induced apoptosis was prevented by 0.8 ng/ml rhEpo and by 10 µM NSB (Fig. 3a; Epo p = 0.044; NSB p = 0.043). Combined treatment with the same concentrations of rhEpo and NSB also increased relative neuron survival in hypoxia-exposed cultures (from 0.81 to 0.96 median relative survival; p = 0.066) to the level of normoxic control cultures, but this increase did not reach significance level. Hypoxia (< 0.3% O_2_; 36 h) increased the expression of *Tc-ace-1* (1.2-fold ± 0.2 SD; p = 0.0039) and *ace-2* (1.33-fold ± 0.3 SD; p = 0.0079) transcripts in *T. castaneum* neurons (Fig. 3b). *Tc-ace* gene expression was normalized to *rps3* and *rps18* whose abundance remained stable during the hypoxic period. Treatment of hypoxia-exposed cultures with neuroprotective concentration of rhEpo prevented the increase of *Tc-ace-1* expression (1.05-fold ± 0.2 SD compared with normoxic control cultures; p = 0.09 to control, p = 0.0039 to hypoxia *ace-1* expression) but not the increase of *Tc-ace-2* expression (1.2 ± 0.1 SD; p = 0.044 to control and p = 0.35 to hypoxia expression) (Fig. 3c). Thus, while hypoxia induces apoptosis and elevated expression of *Tc-ace-1* and *Tc-ace-2* in *T. castaneum* neurons, Epo-mediated neuroprotection correlates with suppressed *ace-1* expression, suggesting that elevated *Tc-ace-2* transcript levels alone are not sufficient to drive apoptosis.

Previous studies reported optimum-type dose–response curves for Epo-mediated protection of mammalian and insect neurons^14,16–19^. However, no mechanistic explanation for toxic effects of high Epo concentrations has been provided so far. In this context, we compared *ace* expression in primary neuron cultures following exposure to neuroprotective and toxic concentrations of rhEpo. Serum was removed from culture media after three days in vitro as a mild apoptogenic stimulus before neurons were stimulated with rhEpo for 48 h starting on day five in vitro. In *T. castaneum* neurons neuroprotective concentrations of rhEpo (0.8 ng/ml) reduced the expression of both *Tc-ace-1* (0.72-fold ± 0.1 SD, p = 0.0095) and *Tc-ace-2* (0.69-fold ± 0.1 SD, p = 0.0095) compared with untreated control cultures (Fig. 3c). Toxic concentrations of rhEpo (8 ng/ml) affected the expression of the two *ace* genes differentially, leading to increased *Tc-ace-1* (1.33-fold ± 0.3 SD, p = 0.035) and decreased *Tc-ace-2* (0.71-fold ± 0.2 SD, p = 0.0095) transcript levels compared with untreated controls (Fig. 3c). *Tc-ace* gene expression was normalized to *rps3* and *rps18* whose abundance was not altered by rhEpo stimulation. For validation of CRLF3-dependant *ace-1* regulation in beetle neurons, we prepared primary neuronal cell cultures and knocked down *CRLF3* expression via RNAi. Figure 3d shows, that *ace-1* is significantly upregulated in hypoxic conditions as expected (1.39 ± 0,14 SD; p = 0.034), while Epo treatment during hypoxia inhibits *ace-1* upregulation (0.95-fold ± 0,07; p = 0.88 to control and p = 0.0034 to hypoxia group). Cells lacking CRLF3, but receiving Epo before hypoxia exposure show significant upregulation of *ace-1*, similar to sole hypoxia-exposed group (fold change 1.28 ± 0.24; p = 0,035 to control, p = 0.89 to hypoxia and p = 0.035 to Epo-treated groups). Knockdown of *CRLF3* expression reduced the hypoxia-induced increase of *ace-2* transcript levels, which was not affected by rhEpo.

All experiments involving treatment with Epo were additionally performed with primary neuron cultures of *L. migratoria* (limited to effects on *Lm-ace-1* since the sequence of *Lm-ace-2* is still unknown) for comparison between different insect species. Supplementary Figure S2 summarizes these data. Briefly and as described for *T. castaneum,* hypoxia-induced neuronal death is prevented by neuroprotective concentration of rhEpo (33.3 ng/ml) and NSB-mediated (10 µM) inhibition of AChE. Hypoxia (36 h) increases *Lm-ace-1* transcript levels which is prevented by rhEpo (33.3 ng/ml). RNAi-mediated knock down of *Lm-crlf3* abolished Epo’s effect on *ace-1* expression in hypoxic conditions. Exposure to 33.3 ng/ml rhEpo has no impact on *Lm-ace-1* transcript levels whereas 333 ng/ml rhEpo (toxic for primary locust neurons^10^) elevates *Lm-ace-1* transcript level by 2.3-fold.

## Discussion

AChE is an important regulator and executor of apoptosis in mammalian cells and altered presence of AChE is associated with various degenerative diseases and cancer^40,42,43,61,62^. A pro-apoptotic function of AChE, that parallels its role in mammals, was recently reported in the migratory locust *L. migratoria*^60^. Due to incomplete genomic information in this species only one (*ace-1*) of typically two genes coding for AChE in insects has so far been identified. Hence, we extended our studies to the beetle *T. castaneum* in which expression and function of both *Tc-ace-1* and *Tc-ace-2* could be differentially studied. Previous studies suggested that *Tc*-AChE-1 is predominantly responsible for ACh hydrolysis at cholinergic synapses while *Tc-*ACh-2 is involved in developmental processes^55^.

In regard to AChE isoforms by alternative splicing and alternative promotor selection in vertebrates^39,63^ and reports about multiple and partly stress-induced AChE-2 splice variants in *Drosophila melanogaster*^53,64^ we explored the possibility of alternatively spliced *T. castaneum ace-1* and *ace-2*. qPCR-based analysis of pupal brains with various pairs of exon-spanning primers detected single transcripts of respective sizes that were expected in the absence of splicing. Detected transcripts were identical in brains of untreated pupae and pupae that were exposed to hypoxia for 24 h. These results suggest a single transcript from the *Tc-ace-2* locus under both normal and physiologically challenging conditions. Since cyclorrhaphan flies possess only one gene for AChE (a paralogue to *ace-2* of other insects) the previously reported alternative splicing of *ace-2* in *D. melanogaster* may be required to generate different types of AChE that perform synaptic and extrasynaptic functions^53^.

Survival of unchallenged and hypoxia-exposed primary neurons from *L. migratoria* increased with pharmacological inhibition of AChE^60^. This indicated a general pro-apoptotic role for AChE in this species that parallels AChE functions in vertebrates. Hypoxia-challenged neurons of *T. castaneum* were also rescued from apoptotic cell death by AChE inhibition (this study), suggesting the general presence of AChE-mediated pro-apoptotic functions in insect neurons and probably other cell types. In contrast to unselective pharmacological inhibition of both AChE-1 and AChE-2 by two different inhibitors (NSB and TRB), which completely prevented hypoxia-induced cell death, RNAi-mediated knockdown of either *Tc-ace-1* or *Tc-ace-2* expression rescued hypoxia-exposed neurons only partially (Fig. 2, Suppl. Fig. S1). Incapability of full rescue may be related to incomplete downregulation of AChE protein, partial functional compensation by the other AChE type or direct contribution of AChE types to some but not all apoptotic mechanisms.

In contrast to *L. migratoria*, sequences of both *ace* genes are available for *T. castaneum*, allowing studies of the differential expression of *Tc-ace-1* and *Tc-ace-2* in this species. Transcript levels of both *ace* genes were elevated in the brains of hypoxia-exposed *T. castaneum* pupae, with more pronounced and more persistently enhanced expression of *Tc-ace-1* compared to *Tc-ace-2* (Fig. 1d). Similarly, enhanced expression of both *Tc-ace-1* and *Tc-ace-2* was also detected in primary brain neurons exposed to 36 h of hypoxia, representing a strong apoptogenic stimulus (Fig. 3c,d). Upregulation of *ACHE* expression under apoptogenic conditions has frequently been reported in mammalian cells and tissues. Here, AChE-S, predominantly involved in synaptic ACh hydrolysis, was typically involved^42,52,65^. *Tc-ace-1* is the predominant AChE associated with synaptic functions in *T. castaneum* while *Tc-ace-2* participates in rather diffusely characterized developmental processes^55,58^. Our results suggest that synaptic *Tc-ace-1* seems to play a more important role for the induction and execution of apoptosis than *Tc-ace-2*, since its expression is induced by hypoxia and toxic concentrations of rhEpo.

Having demonstrated an important regulatory function of AChE in apoptosis of locust and beetle neurons (Ref.^60^; this study) we explored the possibility that Epo/CRLF3-mediated neuroprotection relies on interference with pro-apoptotic functions of AChE. CRLF3 is a phylogenetically (from Cnidaria to humans) conserved cytokine receptor, whose endogenous ligand could not be identified in any species. Based on sequence comparison, CRLF3 is a class I cytokine receptor that shares similarities (e.g. initiates transduction via Janus kinase and STAT signalling) with vertebrate receptors for prolactin, growth hormone, thrombopoietin, and Epo^25,26^. Epo/CRLF3 interaction initiates antiapoptotic mechanisms in locust and beetle neurons^19,33^ that share a number of similar characteristics with Epo-mediated protection of mammalian neurons and other non-hematopoietic cell types (reviews^28,30^. Our previous studies already established the activation of CRLF3 by rhEpo to be responsible for neuroprotection in insects^19,33^. However, the downstream mechanisms by which neuroprotection is mediated remained unanswered. With the presented study we demonstrate that rhEpo-mediated activation of CRLF3 prevents the elevated expression of pro-apoptotic *ace-1* in primary *T. castaneum* (Fig. 3b,d) and *L. migratoria* (see Supplemental Fig. S2) neurons. Though dsRNA exposure for five days in vitro may not completely remove all CRLF3 (turnover periods of CRLF3 are not known) the achieved CRLF3 reduction was sufficient for a significant reduction of Epo’s effect on *ace-1* expression. It can thus be concluded that CRLF3-initiated neuroprotective mechanisms in insects include the prevention of pro-apoptotic AChE-1 production. Though rhEpo did not prevent hypoxia-induced elevation of *ace-2* levels in *T. castaneum* neurons, additional knockdown of *CRLF3* expression kept *ace-2* transcript levels similar to that of untreated control cultures. Since the experiments (shown in Fig. 3d) reproduced previous results of exposure to hypoxia and hypoxia/rhEpo (shown in Fig. 3b) and variation of *ace-2* transcript levels after *CRLF3*-knockdown was low between individual experiments, we believe that the observed effect is genuine though not explainable on the basis of our current knowledge.

Protective concentrations of rhEpo prevented both hypoxia-induced apoptosis and hypoxia-induced upregulation of pro-apoptotic *ace-1* expression in *T. castaneum and L. migratoria* primary neurons (Fig. 3, Suppl. Fig. S2). The neuroprotective effects of rhEpo and pharmacological inhibition of AChE with NSB were similar and combined treatment with both substances had no detectable synergistic additive effect on neuron survival in hypoxia. Our experiments with neuroprotective and toxic concentrations of rhEpo confirm that apoptogenic stimuli induce elevated *ace-1* expression and initiation of protective pathways keep *ace-1* expression on basal or even lower levels. These results indicate that activation of CRLF3 by Epo prevents the elevated expression of AChE-1 under apoptogenic conditions and hence suppresses the induction and/or execution of apoptotic cell death. A direct regulation of AChE expression by Epo signalling has been demonstrated in mammalian erythrocyte progenitor cells that express classical EpoR^66^. Epo-stimulated transduction pathways included activation of the transcription factor GATA-1 which induced *ACHE* transcription and production of erythrocytic AChE-E. In erythrocyte progenitor cells, expression of AChE-E was essential for survival and maturation^66^. Epo has previously been described to activate GATA-binding transcription factors that regulate transcription of target genes in various cell types^67–71^. GATA transcription factors are evolutionary conserved and have been associated with innate immune responses^72^. Keeping this in mind, GATA transcription factors could be candidate transcription factors mediating Epo’s regulatory functions described here. Epo has been described to effect different GATA family members^67–71^ which in return can either activate or inhibit the expression of target genes depending on their identity^67–71^. Bearing this in mind, it is possible that while Epo activates GATA-1, which in return induces expression of AChE-E in hematopoietic cells, it might activate another GATA factor which hinders the expression of pro-apoptotic AChE in other cell types. Whether GATA factors provide the link between CRLF3 activation and apoptosis-suppressing restriction of *ace* transcription in insect neurons has to be demonstrated in future studies.

Cell-protective concentrations of Epo depend on cell type, species, physiological condition of the cell and the type of insult in both mammals and insects^10–13,19^. Maximal Epo-mediated protection of primary brain neurons is achieved with 33.3 ng/ml (compares to ~ 4 U/ml) in *L. migratoria* and 0.8 ng/ml (compares to ~ 0.1 U/ml) in *T. castaneum*^10^. Instead of reaching a state of saturation, higher than optimum concentrations of Epo elicit toxic effects. Optimum-type concentration dependence of protection including toxic effects of high concentrations has been reported in mammalian and insect neurons^14–19^. The switch from protective via less protective to toxic effects of further elevated concentrations of Epo could not be explained in the above-mentioned reports. Previous studies identified 333 ng/ml (*L. migratoria*) and 8 ng/ml (*T. castaneum*) as toxic Epo concentrations that reduced the survival of primary brain neurons to ~ 80% compared to untreated control cultures^10^. In the present study, toxic concentrations of Epo increased *ace-1* transcripts in both species while respective neuroprotective concentrations caused reduced (*T. castaneum*) or no alteration (*L. migratoria*) in *ace* expression (Fig. 3, Suppl. Fig. S2). While protective concentration of Epo reduced *ace-2* expression in serum-deprived neurons (Fig. 3c), protective concentration of Epo did not reduce *ace-2* expression in hypoxia-exposed neurons (Fig. 3b) and toxic concentrations of Epo had no effect on *ace-2* expression in serum deprived neurons (Fig. 3c). Depending on the type of apoptogenic stimulus either both *ace* genes were simultaneously regulated by Epo or only *ace-1* expression was affected. Though we cannot explain this observation on the basis of our present knowledge, different challenging stimuli likely elicit different apoptosis-promoting processes which may include *ace-1* and/or *ace-2* and/or additional factors and hence different steps that can be regulated by Epo/CRLF3-activation.

The data from both species studied suggest that proapoptotic *ace-1* is differentially regulated by optimal neuroprotective concentrations and higher toxic concentrations of Epo. How insect neurons distinguish neuroprotective from toxic concentrations of CRLF3 ligand is currently unknown. Whether neurotoxic effects are mediated via (over-)stimulation of CRLF3 or via contribution of another, yet unidentified mechanism remains to be studied.

The data presented in this manuscript clearly identify AChE as a major driver of apoptosis in insect neurons. Apoptogenic stimuli (hypoxia, toxic concentrations of Epo) increase *ace* expression and induce cell death. Activation of CRLF3 (by Epo, as previously established) mediates neuroprotection by preventing the increased expression of pro-apoptotic AChE with larger impact on *ace-1* than on *ace-2*. Studies on primary brain neurons from two insect species (*L. migratoria* and *T. castaneum*) belonging to different taxonomic groups (Orthoptera and Coleoptera) led to similar results, suggesting that the observed mechanisms may be representative for insects. Since both apoptosis and CRLF3 are conserved throughout the animal kingdom, the processes observed in insect neurons may also be present in other cell types and other non-insect species. Altogether, this manuscript suggests the connection between vertebrate and insect Epo-mediated mechanisms and is, to our knowledge, the first report of a connection between AChE and Epo in the regulation of apoptotic mechanisms. Given that many of the results collected in insects on Epo functions could be replicated in mammalian cells and vice versa, it is likely that a similar mechanism is involved in mammalian Epo-mediated cell protection.

## Methods

Experiments were performed with *Tribolium castaneum* late pupae (San Bernadino wildtype strain) kindly provided by the lab of Prof. Dr. Gregor Bucher and *Locusta migratoria* fifth instar nymphs obtained from a commercial breeder (HW-Terra, Herzogenaurach, Germany). Beetles were reared in plastic boxes filled with whole grain flour and yeast at 27 °C, 40% humidity and 12/12 h day/night cycle. Locusts were kept at 24 °C, 55% at 12/12 h day/night cycle.

### Insect primary brain cell culture

Primary neuron cultures were established as previously described^19,32,33,60,73^. In brief, 20 tribolium or 2 locust brains per culture were dissected and collected in Leibowitz 15 medium (Gibco; Life Technologies, Darmstadt, Germany) supplemented with 1% penicillin/streptomycin and 1% amphotericin B (both Sigma-Aldrich, Munich, Germany) (from now referred to as L15 medium). Subsequently, brains were enzymatically digested in collagenase/dispase (2 mg/ml, Sigma-Aldrich, Munich, Germany) for 45 min (*T. castaneum*) or 30 min (*L. migratoria*) at 27 °C. Enzymatic reaction was stopped by repeated washing in Hanks’ balanced salt solution and brains were mechanically dissociated by repeated pipetting in L15. The suspension of dissociated brain cells was seeded on Concanavalin A (Sigma-Aldrich, Munich, Germany) coated coverslips and let to rest for 2 h. Afterwards, culture dishes were filled with L15 supplemented with 5% fetal bovine serum gold (FBSG, PAA Laboratories GmbH, Pasching, Austria). Medium was replaced by L15 plus FBSG on day two and by L15 without serum on day four in vitro. Primary cell cultures were maintained at 27 °C without CO_2_ buffering.

### Pharmacological treatment and hypoxia exposure of primary cell cultures

Cell cultures were treated with 10 µM neostigmine bromide (NSB; Sigma-Aldrich, Munich, Germany), 10 µM territrem B (TRB; initially dissolved in methanol, further diluted in L15; Abcam, Cambridge, United Kingdom) or recombinant human Epo (33.3 ng/ml or 333 ng/ml for locust cultures and 0.8 ng/ml or 8 ng/ml for beetle neurons; NeoRecormon; Roche, Welwyn Garden City, United Kingdom). AChE inhibitors NSB and TRB were applied throughout the entire culturing period and replaced with each medium change. rhEpo was added to the medium on day 5 in vitro. 12 h after the onset of rhEpo treatment, cultures were exposed to hypoxia (< 0.3% O_2_, Hypoxia Chamber; Stemcell, Cologne, Germany) for 36 h. Untreated control cultures were maintained at normoxic conditions. Subsequently cell cultures were fixed and stained as described below. To compare effects of protective and deleterious concentrations of rhEpo, cultures were exposed to different concentrations of rhEpo for 48 h starting on day five in vitro.

### RNA interference with ace-1 and ace-2 expression in *T. castaneum* neurons

Double-stranded (ds) RNA fragments targeting *Tc-ace-1*,*Tc-ace-2, Tc-crlf3* or *Lm-crlf3* were designed and prepared as stated below (Both CRLF3 constructs have been published previously by our group^19,33^). To reduce expression of respective protein, 10 ng/ml dsRNA targeting either one of the genes was added from the beginning of the experiment and renewed with each medium change. Successful interference with protein expression by “soaking RNAi” was previously demonstrated in primary cultured *T. castaneum* neurons^19,73^. Cells were exposed to hypoxia on day 5 for 36 h (O_2_ < 0.3%) before being fixed and analyzed for cell survival.

### DAPI staining and analysis of cell survival

After treatments, cells were fixed in 4% paraformaldehyde (PFA) for 30 min. Cells were washed 3 times in phosphate-buffered saline (PBS) and twice in PBS/0.1% Triton-X-100 (PBST) for each 5 min. Subsequently nuclei were stained with DAPI (Sigma-Aldrich, Munich, Germany 1:1000 in PBST) for 30 min before being washed 5 times in PBS. Coverslips were transferred to microscopy slides, enclosed in DABCO (Roth, Karlsruhe, Germany) and sealed around the edges with nail polish.

**Figure 4:**
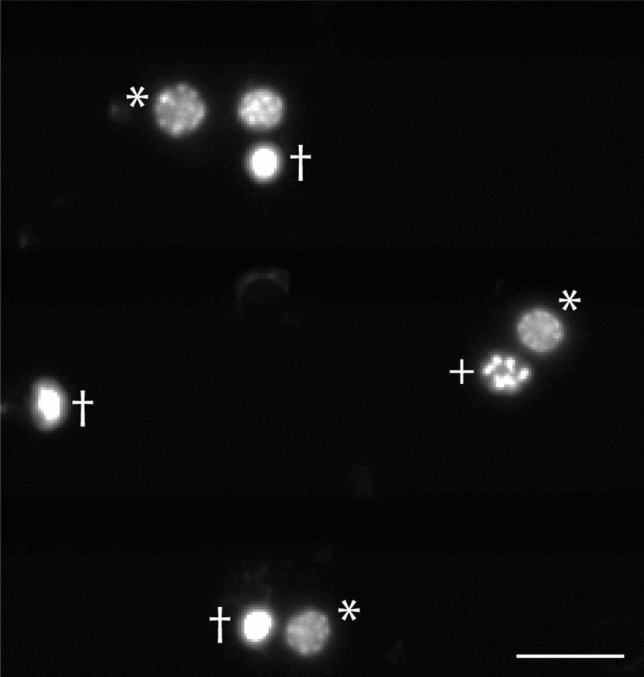
DAPI stained *T. castaneum* primary neurons. Cell survival was analysed by automatic counting nuclei of intact (*), dying (+) and dead (†) neurons. Scale bar 10 µm.

Images of DAPI-stained nuclei were taken using an epifluorescence microscope (Zeiss Axioskop, Oberkochen, Germany) equipped with a spot CCD camera (Invisitron, Puchheim, Germany). From each cell culture, two rows of non-overlapping photographs were taken (for locust cultures ~ 80 images using × 40 magnification; for *tribolium* cultures ~ 120 images using × 63 magnification). Cell survival was assessed by the DAPI stained chromatin structure as described previously^19,32,33,60,73^. In brief, alive and dead/dying cells can be identified by chromatin condensation, which is represented by increasingly uniform and intense DAPI staining of nuclei (see Fig. 4). The intact and dead/dying neurons were identified and counted automatically by an object recognition AI based on the Faster R-CNN^74^ net with Inception V2^75^. The AI was trained by us to categorize living and dead cells for *Locusta* (trained by 3480 cells in 92 images) and *Tribolium* (trained by 3469 cells in 75 images). We re-trained a neuronal net that was previously trained and configured for the Oxford-III Pets dataset^76^ and is available as part of the tensor-flow model collection^77^. All routines for the AI cell counting routines were written in Python 3.5^78^ utilizing numpy^79^, pandas^80^, and tensor flow^81^ amongst others.

Cell survival in different treatment groups within one experiment was subsequently normalized towards the corresponding untreated control, set to 1. Morphological changes of DAPI-labelled chromatin have previously been correlated with the induction of specific markers for apoptosis, including TUNEL staining and caspase activation^32,60^.

### dsRNA cloning and preparation

Two non-overlapping fragments targeting either *Tc-ace-1* (HQ260968) *or Tc-ace-2* (HQ260969) were designed and cloned into the pCRII vector by TA cloning (TA cloning Kit, Invitrogen, Life Technologies, Darmstadt, Germany) (Fragment sequences are listed in Supplements). Vectors were transformed into XL-1 blue competent cells and grown on ampicillin-supplemented agar plates. Multiple clones were analyzed by colony PCR and sequencing for the proper insertion of the target fragments. Clones with the appropriate vector were grown and DNA was extracted using NucleoSpin Plasmid Kit (Macherey–Nagel, Düren, Germany).

For dsRNA preparation, plasmids were amplified by PCR using M13 fwd and M13 rev primers with a T7 RNA promoter sequence attached to the reverse primer. The PCR program and primer sequences are listed in Tables 1 and 2. PCR products were separated on a 1% agarose gel and purified using the Macherey–Nagel NucleoSpin Gel and PCR Clean-up Kit (Macherey–Nagel, Düren, Germany) according to the manufacturer’s recommendations.

**Table 1:** Oligonucleotides used in this study.

	Sequence 5ʹ–3ʹ	Amplicon (bp)
M13 fwd	GTAAAACGACGGCCAGT	300
M13-T7 rev	TAATACGACTCATAGGCAGGAAACAGCTATGAC	
Tc *ace-2-*E1-E3 fwd	GCCAGAGACTTTCACAGCGA	1177/359
Tc *ace-2-*E1-E3 rev	CATCACGTTCCAACCGACTC	
Tc *ace-2*-E2-E4 fwd	CGGCTTCCTCTACTTGAGCA	731/588
Tc *ace-2*-E2-E4 rev	TCTGGTTCAAGTAGCCGTCG	
Tc *ace-2-*E3-E5 fwd	GAGTCGGTTGGAACGTGATG	345/192
Tc *ace-2-*E3-E5 rev	GCTGCAAATCTGGCAAAGGC	
Tc *ace-2*-E4-E6 fwd	CGACGGCTACTTGAACCAGA	424/266
Tc *ace-2*-E4-E6 rev	ATCGTTCCAAAACGCGCACG	
Tc *ace-2*-E4-E7 fwd	CGACGGCTACTTGAACCAGA	534/376
Tc *ace-2*-E4-E7 rev	TGCTCAAGTAGAGGAAGCCG	
Tc *ace-1* fwd	AACTTCAGCAGCAAACGAGC	120
Tc *ace-1* rev	CTGTCGACACCATCAGGAGG	
Tc *ace-2* fwd	ACAGCTGAGGTTCAGGAAGC	116
Tc *ace-2* rev	GGGAAGTACTCGTAGCGCTC	
Tc *rps3* fwd	GGCGCTAAAGGGTGTGAAGT	150
Tc *rps3* rev	TGTCTTAGCAAGACGTGGCG	
Tc *rps18* fwd	CCTCAACAGGCAGAAGGACA	130
Tc *rps18* rev	CCTGTGGGCCCTGATTTTCT	
Lm *ace-1* fwd	TTTGAAATGGCGGTGGTAGC	120
Lm *ace-1* rev	GTCGGAGGACTGCCTGTAC	
Lm *18s rRna fwd*	CATGTCTCAGTACAAGCCGC	106
Lm *18s rRna rev*	TCGGGACTCTGTTTGCATGT	
Lm *gapdh* fwd	GTCTGATGACAACAGTGCAT	110
Lm *gapdh* rev	GTCCATCACGCCACAACTTTC	

Primers for M13, M13-T7, Lm ace-1, 18srRNA and gapdh were previously used^19,60^.

Oligonucleotides used for splice variant analysis of Tc-ace-2 may potentially generate two amplicons.

*Lm L. migratoria*, *Tc T. castaneum*.

**Table 2:** PCR program for dsRNA template amplification.

Step	Temperature (°C)	Time (s)	Cycle
Initial denaturation	98	180	
Denaturation	98	30	× 30
Annealing	60	30	
Elongation	72	30	
Final elongation	72	300	

Purified DNA was subsequently in vitro transcribed by usage of the MEGAScript T7 transcription kit (Life Technologies, Darmstadt, Germany) following the manufacturer’s instructions. The single-stranded RNA was washed three times in 70% EtOH before resuspension in injection buffer (1.4 mM NaCl, 0.07 mM Na_2_HPO_4_, 0.03 mM KH_2_PO_4_, 4 mM KCl). Single-stranded RNA was annealed to double strands (dsRNA) at 94 °C for 5 min and cooled down to 20 °C at a rate of 0.1 °C per second. dsRNA concentrations were measured with a spectrophotometer (Nanodrop 1000, Thermo Fisher Scientific, Schwerte, Germany). dsRNA quality was assessed by agarose gel electrophoresis.

### RNA isolation and cDNA synthesis

RNA of cell cultures and brains was isolated using Trizole (Thermo Fisher Scientific, Schwerte, Germany) as described previously^60,73^. For tissue specimen 15 brains of late *T. castaneum* pupae were extracted and collected in RNALater (Sigma-Aldrich, Munich, Germany). In the case of cell culture specimen, 5 cultures of each treatment group were prepared as described above. Cells were scraped in medium and cell suspension was centrifuged at 21.000×*g* for 5 min. Medium was discarded and the cell pellet was washed in PBS once before RNA isolation.

In brief, 1 ml Trizole was added per sample and samples were homogenized using a tissue lyser (Qiagen, Hilden, Germany) at 50 Hz for 3 min (stainless steel beads were used in case of tissue samples). Subsequently 200 µl chloroform (Labsolute, Th. Geyer, Renningen, Germany) was added and the mixture was returned into the tissue lyser for 20 s. Samples were centrifuged at 12.000×*g* for 15 min at 4 °C and the translucent, RNA-containing phase was carefully transferred to a fresh Eppendorf tube and mixed with 1 ml ice cold 70% EtOH. Tissue samples were incubated for at least 30 min at − 20 °C. Cell culture samples were incubated overnight. The precipitated RNA was centrifuged at 10.000×*g* for 15 min at 4 °C and the RNA pellet was washed three times in ice cold 70% EtOH. RNA pellets were air dried and resuspended in 6–30 µl ddH_2_O. RNA concentrations were measured with a spectrophotometer (Nanodrop 1000, Thermo Fisher Scientific, Schwerte, Germany).

Complementary DNA (cDNA) was synthesized using the NEB LunaScript RT SuperMix Kit (New England BioLabs, Ipswich, MA, USA) according to the manufacturer’s instructions.

### Ace splice variant analysis

In order to identify if *Tribolium* performed alternative splicing on *ace-2,* exon spanning primers, skipping one exon, were designed (see Table 1). Primers were set into the middle of each exon and reverse transcription PCR (RT-PCR) from brain cDNA was run. RNA and cDNA were prepared as described above. RT-PCR Program can be seen in Table 3. RT-PCRs were performed using GoTaq Green Master Mix (Promega, Madison, USA) according to the manufacturer’s instructions. Expected amplicon sizes in case of alternative splicing can be seen in Table 1.

**Table 3:** RT-PCR program for Tc *ace-2* splice variant analysis.

Step	Temperature (°)	Time (s)	Cycle
Initial denaturation	98	180	
Denaturation	98	30	× 30
Annealing	61	30	
Elongation	72	30	
Final elongation	72	300	

### qPCR analysis for *Tc-ace-1* and *Tc-ace-2* expression in vitro and in vivo

In order to evaluate if either *Tc-ace-1* or *Tc-ace-2* are differentially expressed in neurons of *T. castaneum* pupae during physiological stress qPCR analyses were performed.

*Tribolium castaneum* pupae were exposed to hypoxia for either 24 or 36 h. Control animals remained in normoxic conditions. 15 brains were extracted and collected in RNALater. RNA isolation and cDNA synthesis were performed as described above. For cell culture experiments cells of different treatment conditions (normoxia, hypoxia, rhEpo, rhEpo + RNAi) were collected and prepared as described above.

qPCR analysis was run using primers for amplification of *T. castaneum ace-1* and *ace-2* and *L. migratoria ace-1* (EU231603). *Rps18* and *rps3* (TC014405 and TC008261) were run as controls for beetle neurons, while *18s rRna* and *gapdh* (AF370793 and JF915526) were used for qPCR analysis of locust neurons. Prior to experimental qPCR runs, all primers for housekeeping genes were tested for efficiency and stability in hypoxic conditions. Primer sequences are listed in Table 1.

qPCRs were run using the MyiQ™ Single-ColorReal-Time PCR Detection System (Bio-Rad, Munich, Germany) in a sealed 96-well plate. Final PCR reactions contained 5 µl Luna Universal qRT-PCR Master Mix (New England Bio- Labs, Ipswitch, MA, USA), 0.1 µM forward and reverse primers and 10 ng cDNA resulting in a final reaction volume of 10 µl. All samples were run as triplicates and (−) RT and water controls were always included. The PCR amplification protocol is displayed in Table 4.

**Table 4:** qPCR program employed for gene expression studies.

	Step	Temperature (°C)	Time (s)	
PCR reaction	Initial denaturation	95	180	
	Denaturation	95	10	× 40
	Annealing	61	30	
	Elongation	72	30	
Melting curve	Denaturation	95	60	
	Annealing	55	60	
	Melting curve	55	10	0.5 °C per cycle up to 95 °C

Data was analyzed using the Pfaffl method^82^ and geometric means of both housekeeping genes were calculated and normalized towards the control group for both species.

### Statistical analysis and data plotting

All statistical calculations were performed with RStudio (Version 1.2.1335). Pairwise permutation tests (two-tailed) contained in the R packages “coin” and “rcompanion” were employed and combined with Benjamini–Hochberg corrections for multiple comparisons^83,84^. Normalized relative survival data are plotted as box plots, depicting the median cell survival, upper and lower quartile and whiskers representing 1.5× interquartile ranges. Dots represent data points from individual experiments. qPCR results are shown as bar plots of geometric mean calculations of single experimental data. Standard deviations were calculated with Excel (Microsoft).

## Supplementary Information

Supplementary Information 1.

Supplementary Information 2.

## Data Availability

All raw data published in this manuscript can be accessed on reasonable request. All data forming the basis of quantifying graphs are provided as Supplement.
