# Supplementary material for: Protection of insect neurons by erythropoietin/CRLF3-mediated regulation of pro-apoptotic acetylcholinesterase
Source: Sci Rep. 2022 Nov 3;12:18565. doi: 10.1038/s41598-022-22035-0 (PMC9633726; doi:10.1038/s41598-022-22035-0)
Supplement: Supplementary file 1 — Supplementary Information 1. [file 41598_2022_22035_MOESM1_ESM.docx]

Supplementary Data

*Tribolium ace-1* coding sequence. Fragment 1 for RNAi mediated knockdown is marked green, Fragment 2 is marked in blue.

caacgaccgttgtgcaaatacattaaatccaaacaagtgtttaaatcatcggccattccgtttatttataggccgctttgttgcagaattattgaaaagcgatgacgggagcgtgggcggcctgcttactagtgattctacttccctcatgcatcccttcgccacaccgaggccgccaccacccccccgagcctcacgccgaggcctaccacatgtcccgcgaccccttcgacccgcaccgggactcggaggagttccgacgagacgccccggacgacaagcgggagtttacgcgtcgcgactccgaagatgatcccctggtcatccagacaaagaaagggaaagtccgcggcatctccctcacggccgccacgggaaagaaggtcgacgcgtggctgggaatcccctacgcgcagaaaccgctcggcaacctccggttccggcacccgcggcctgcggaaaagtgggagggcgtgatgaacacgactagccagccgaattcctgcgtgcagattatagacacagtgtttggggatttcccaggtgcgaccatgtggaaccccaacacaccgttgaacgaagactgtctttacgtgaacgtggtggtgccgaagccgaggccgaccagtgccgcggtcatggtgtgggtgttcgggggtgggttctactccgggacgaacactttagaagtttacgaccacaacatactagtgtccgaagagaacattattttagtttcgatgcagtatagagttgcttcgctcggattcctgtatttcgggacgccggacgttccgggaaatgcaggattgtttgaccaaatgatggcgttgcaatgggtacgtgataacatcgccgctttcggaggaaaccccaacaatatcactctattcggagaatcagcaggagctgtttcagtctccctgcatctactctctccattatcgagaaacctattctcacaagctatcatggaatctgggagtgcgacagcaccttgggctataatctcccgtgaagaaagcattttgcgaggattgagactagcagaagctgtgggttgtccgcatgagcgccacgagctttccgctgttatagactgcttaaaaaagaaagaccctattgatttagtcaataacgaatggggaacactcggcatatgtgagtctccgttcgtcccagtcattgatggagcctttctggacgaatcccccacacgggctctggcaaacaaaaacttcaaaaaaaccaatatcctcatgggttctaacacagaagaaggctactacttcataatctactacttgaccgaactgttccgaaaggaggagaacgtttacgtcaaccggcaggaattcctgcgggctgtaaccgaactgaacccgtacttcaacgcgatttcgcgccaagcaatcgtcttcgaatacaccaactggttaaaccccgacgacccggtgagcaaccgcgactccctcgataaaatggtaggcgactaccacttcacttgcaacgtcaacgaattcgcccaccggtacgccgaaaccggcaacacggtctacatgtactactacaagcaccggacagtggcgaatccctggccctcctggaccggcgtgatgcacgccgacgaaatcaactacgtcttcggggaaccactcaatcccactaaaagccacacagcacaagaagtcgatctgagtaagcgaatcatgagatactgggccaatttcgccaagacgggcaacccgagtcagtcgccgaacggcgtttggacacccactttctggcccccgcacacagctttcggaagggagtttctcaccctcgatgtcaactccactgccacagggaggggacctagactcaagcagtgtgccttctggaagaaatacctacctcaacttcagcagcaaacgagcgagcttctgaaccaaccgccccggcaaaactgtaccgacgcggccagttcgttgcgatggtcaagagacggcgctgccggcctcctgatggtgtcgacagtggcggcgctcctcgcggggcctttctaacttagagaaaagtccgtttaggagttcccctgaagcgcattagaaactagcgccttgaaacatatctctagtattgtgtgtgaggtattcaaaatcaacacgac

Fragment 1: tcggaggaaaccccaacaatatcactctattcggagaatcagcaggagctgtttcagtctccctgcatctactctctccattatcgagaaacctattctcacaagctatcatggaatctgggagtgcgacagcaccttgggctataatctcccgtgaagaaagcattttgcgaggattgagactagcagaagctgtgggttgtccgcatgagcgccacgagctttccgctgttatagactgcttaaaaaagaaagaccctattgatttagtcaataacgaatggggaacactcggcatat

Fragment 2: cccgtacttcaacgcgatttcgcgccaagcaatcgtcttcgaatacaccaactggttaaaccccgacgacccggtgagcaaccgcgactccctcgataaaatggtaggcgactaccacttcacttgcaacgtcaacgaattcgcccaccggtacgccgaaaccggcaacacggtctacatgtactactacaagcaccggacagtggcgaatccctggccctcctggaccggcgtgatgcacgccgacgaaatcaactacgtcttcggggaaccactcaatcccactaaaagccacacagc

*Tribolium ace-2* coding sequence. Fragment 1 for RNAi mediated knockdown is marked green, Fragment 2 is marked in blue.

ATGGGTTCGAATCTCGTCGTCGTCGTGGTCGTCGTGGTGGTGGTGGTGGCGTCGCTGAGTGCTTCCGTCCGCGCCTACTCCTGGCCCAGCGAGGAGACGACGACGCGTCCGCCCCAAGCCAGAGACTTTCACAGCGACCCGCTGGTGGTGGAGACCACCAGCGGCCTGGCGCGGGGCAAGGCCAAGACGGTGCTGGGCCGCGAGGTGCACGTCTTCACCGGGATTCCCTTCGCCAAGCCGCCCATCGAACAGCTGAGGTTCAGGAAGCCCGTGCCCATCGATCCCTGGCACGGCACCTTAGATGCCACCAAGCTGCCCAACTCGTGCTACCAGGAGCGCTACGAGTACTTCCCCGGCTTTGAGGGCGAGGAGATGTGGAACCCCAACACGAACATCTCCGAGGACTGCCTCTACCTCAACATCTGGGTGCCCCAGCGCTTGCGCATCCGGCACCACGGCGAGAAGCTCCCCCAGGACCGGCCCAAAGTCCCCGTCCTCGTGTGGATCTACGGCGGGGGCTACATGAGCGGCACCTCCACCCTGGACATCTACGACGCCGACATCATCGCCGCCACCTCCGACGTCATCGTCGCCTCCATGCAATATCGCGTGGGGGCGTTCGGCTTCCTCTACTTGAGCAAGTACTTCCCTCGCGGCAGTGAGGAGGCGCCCGGTAACATGGGCATGTGGGACCAAGCCCTGGCCATCCGCTGGATCAAGGAGAACGCGGCGGCCTTTGGGGGCGACCCAGACCTCATCACGCTGTTTGGGGAGTCGGCTGGAGGCGGCTCCGTCAGCATCCTGCTCCTGAGTCCGGTTACTAAAGGCCTGGCCAGGAGGGGGATTCTGCAGTCGGGGACTATGAACGCCCCTTGGAGTTACATGTCGGGGGAGAGGGCGCCGCAAATCGGGAAGGTCCTGGTGGAGGACTGCGGGTGCAACGTCTCCTTGTTGGAGACGAGGCCGCATGAGGTCATTGATTGCATGAGGGCGGTGGAGGCCAAGACGATTTCGCTGCAACAGTGGAATTCGTATTCGGGGATTTTGGGCTTCCCCTCAACGCCTACGGTTGATGGCGTCTTCATGCCCAAGCATCCCATGGATATGCTGGCGGAAGGGGATTACGAGGATATGGAGATCCTGGTCGGGAGTAACCAAGATGAAGGCACTTACTTCTTACTTTACGATTTTATCGATTTCTTCGAAAAGGATGGCCCTAGCTTCCTCCAACGAGACAAATACCACGACATTATCGATACGATATTCAAAAATATGAGTCGGTTGGAACGTGATGCCATAGTATTTCAGTATACTGATTGGGAGCACGTCAACGACGGCTACTTGAACCAGAAAATGGTGGGCGACGTCGTCGGAGATTATTTTTTCATTTGTCCAACCAACGATTTCGCCGAGCTGGCAGCAGAGCGCGGAATGAAAGTCTACTATTATTTTTTCACACACAGGACAAGCACGTCGTTGTGGGGCGAATGGATGGGGGTGATGCACGGGGATGAGATAGAATACGTGTTTGGCCATCCTTTGAACATGTCGTTGCAGTTTAACTCAAGGGAACGGGAACTCAGTCTGAAGATAATGCAAGCCTTTGCCAGATTTGCAGCAACGGGGAAACCAGTGACAGACGACGTGAATTGGCCATTGTACATAAAAGACCAACCGCAGTATTTCATCTTCAACGCCGACAAAAACGGCATCGGCAAAGGTCCTCGAGCGACAGCGTGCGCGTTTTGGAACGATTTCCTGCCCAAGCTTCGGGATAACCCAGGTAAATTCAATCGTTGCAGTCTGCATCATTAA

Fragment 1: GTCTTCACCGGGATTCCCTTCGCCAAGCCGCCCATCGAACAGCTGAGGTTCAGGAAGCCCGTGCCCATCGATCCCTGGCACGGCACCTTAGATGCCACCAAGCTGCCCAACTCGTGCTACCAGGAGCGCTACGAGTACTTCCCCGGCTTTGAGGGCGAGGAGATGTGGAACCCCAACACGAACATCTCCGAGGACTGCCTCTACCTCAACATCTGGGTGCCCCAGCGCTTGCGCATCCGGCACCACGGCGAGAAG

Fragment 2: CCTCCATGCAATATCGCGTGGGGGCGTTCGGCTTCCTCTACTTGAGCAAGTACTTCCCTCGCGGCAGTGAGGAGGCGCCCGGTAACATGGGCATGTGGGACCAAGCCCTGGCCATCCGCTGGATCAAGGAGAACGCGGCGGCCTTTGGGGGCGACCCAGACCTCATCACGCTGTTTGGGGAGTCGGCTGGAGGCGGCTCCGTCAGCATCCTGCTCCTGAGTCCGGTTACTAAAGGCCTGGCCAGGAGGGGGATTC


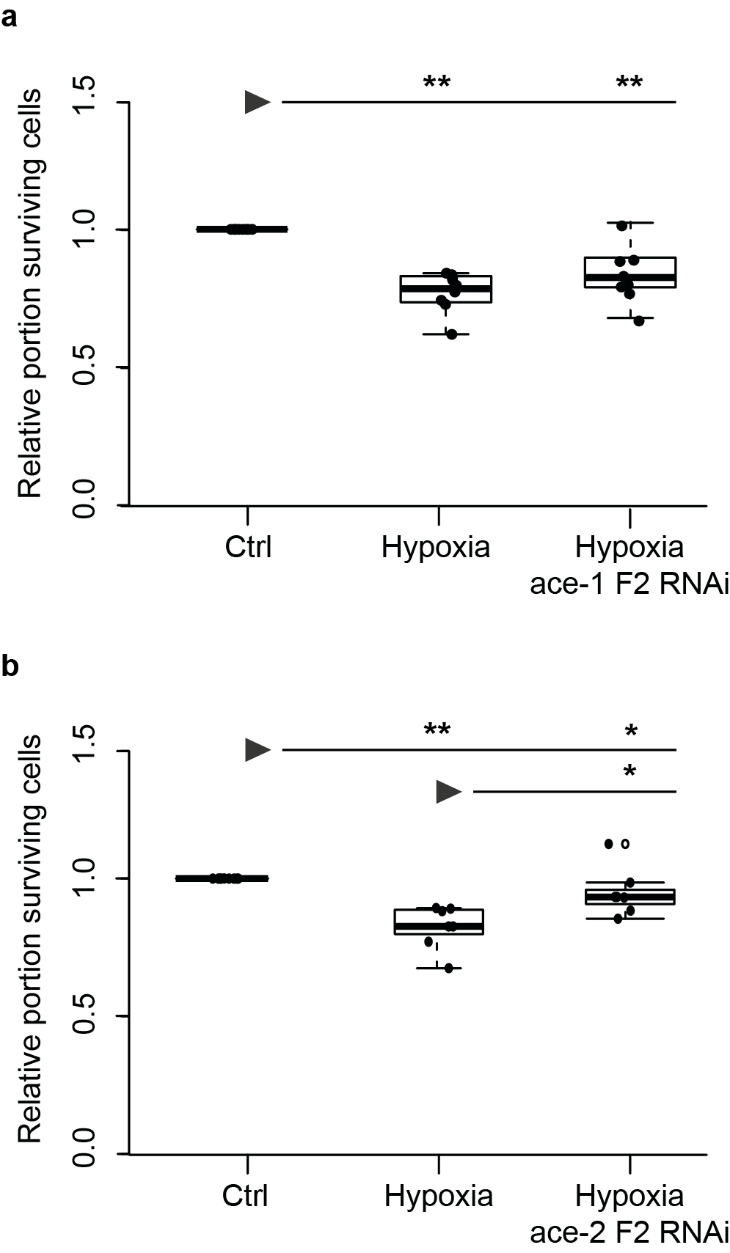


**Supplementary Fig 1** RNAi mediated knock down of Tc-ace-1 and Tc ace-2 in T. castaneum primary cell cultures. **a:** Knock down using Tc ace-1 Fragment 2 (F2) did not rescue cells from hypoxia-induced apoptosis. n=8, 93919 cells analyzed. **b**: Knock down of Tc ace-2 using Fragment 2 (F2) significantly increased cell survival of hypoxia-challenged neurons in comparison to sole hypoxia exposure. Cell survival is however yet significantly reduced in comparison to control cultures. n=7, 83710 cells analyzed. Pairwise permutation test with Benjamini-Hochberg correction for multiple comparison. Significances shown by asterisks (* p<0,5; ** p<0,01)


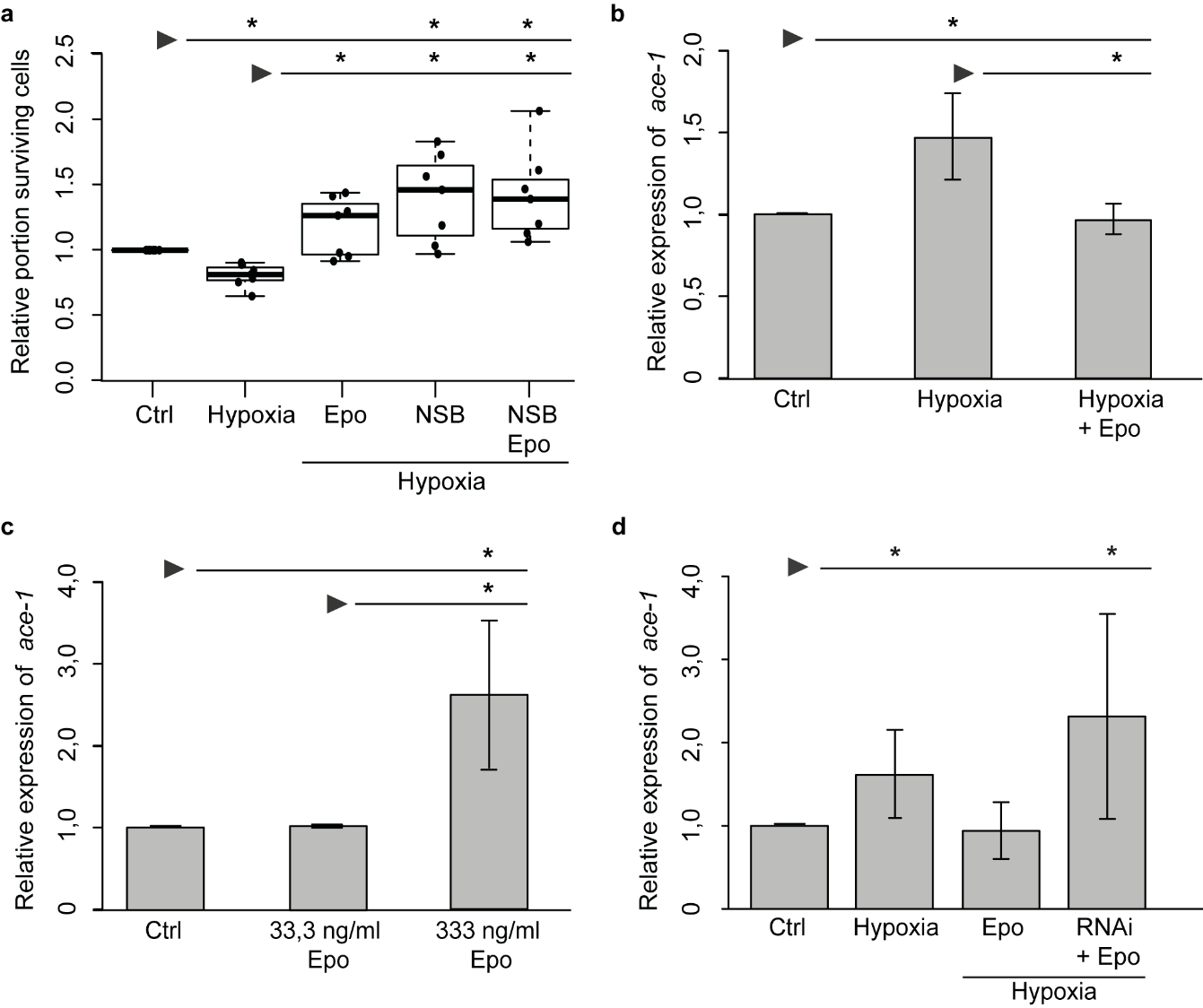


**Supplementary Fig 2: rhEpo regulates cell survival by *ace-1* expression in locust primary neurons.** Cultures were exposed to hypoxic conditions (<0,3% O_2_ for 36 h) and treated with 10 µM NSB (entire in vitro period) or/and 33,3 ng/ml rhEpo (starting 12 h before start of hypoxic period). **a:** Relative survival of primary neurons normalized to untreated normoxic controls. Hypoxia significantly decreased neuron survival. rhEpo, NSB and rhEpo/NSB prevent hypoxia-induced cell death and increase survival at least to the level of normoxic controls. n=7, 56845 cells analyzed. **b:** qPCR analysis of Lm-*ace-1* expression in primary neuron cultures. Hypoxia increases *ace-1* expression (1,47 ± 0,3 *SD*). Hypoxia-induced increase of Lm-*ace-1* transcript levels is prevented by rhEpo (0,96 ± 0,1 *SD*). n=4. **c:** L. migratoria: 33,3 ng/ml Epo (= protective concentration) has no impact on Lm-*ace-1* transcript levels. 333 ng/ml rhEpo (= toxic concentration) significantly increases *ace-1* expression to 2,3-fold (± 0,8 *SD*) compared with untreated controls. n=4. **d:** RNAi-mediated knock down of CRLF3 alters the gene expression response of locust neurons to Epo in hypoxic conditions. Hypoxia increases *ace-1* expression significantly in comparison to control cultures (1,61 ± 0,5299 SD), while Epo treatment (33,3 ng/ml for 12 h before hypoxia onset) retains *ace-1* transcripts at control levels (0,94 ± 0,34 SD). Cells that lack CRLF3 (RNAi) show significantly increased *ace-1* expression after Epo treatment in hypoxic conditions (2,31 ± 1,2 SD). n=4.Statistics with pairwise permutation test and Benjamini-Hochberg correction. Significant differences are indicated by asterisks (* p<0,5; ** p<0,01).
